# Long non‐coding RNA NEAT1 serves as a novel biomarker for treatment response and survival profiles via microRNA‐125a in multiple myeloma

**DOI:** 10.1002/jcla.23399

**Published:** 2020-07-01

**Authors:** Haifeng Yu, Shuailing Peng, Xi Chen, Shuiyun Han, Jialin Luo

**Affiliations:** ^1^ Department of Lymphatic Medical Oncology Cancer Hospital of the University of Chinese Academy of Sciences（Zhejiang Cancer Hospital） Hangzhou China; ^2^ Department of Lymphatic Medical Oncology Institute of Cancer and Basic Medicine（IBMC） Chinese Academy of Sciences Hangzhou China; ^3^ Department of Radiotherapy Cancer Hospital of the University of Chinese Academy of Sciences（Zhejiang Cancer Hospital） Hangzhou China

**Keywords:** long non‐coding RNA NEAT1, microRNA‐125a, multiple myeloma, survival, treatment response

## Abstract

**Background:**

The present study aimed to explore the association of long non‐coding RNA nuclear paraspeckle assembly transcript 1 (lncRNA NEAT1) with multiple myeloma (MM) risk and further investigate its correlation with clinical features, treatment response, survival profiles, and its interaction with microRNA‐125a (miR‐125a) in MM patients.

**Methods:**

Totally, 114 de novo symptomatic MM patients and 30 healthy donors (as controls) were recruited. Their bone marrow samples were collected before treatment (MM patients) and at enrollment (healthy donors), respectively. Subsequently, plasma cells were isolated from bone marrow for detection of lncRNA NEAT1 and miR‐125a expression via reverse transcription quantitative polymerase chain reaction.

**Results:**

lncRNA NEAT1 was upregulated in MM patients compared with healthy donors and presented with excellent value in distinguishing MM patients from healthy donors. In MM patients, lncRNA NEAT1 positively associated with International Staging System (ISS) stage, beta‐2 microglobulin (β2‐MG), and lactate dehydrogenase (LDH), but not correlated with core cytogenetics and other clinical features. Furthermore, lncRNA NEAT1 negatively associated with complete remission (CR), overall remission rate (ORR), progression‐free survival (PFS), and overall survival (OS). Moreover, lncRNA NEAT1 negatively associated with miR‐125a in MM patients. MiR‐125a was downregulated in MM patients compared with healthy donors, and it negatively associated with ISS stage, β2‐MG, and LDH, but positively correlated with CR, ORR, PFS, and OS in MM patients.

**Conclusion:**

lncRNA NEAT1 might interact with miR‐125a, and serves as a novel biomarker for treatment response and survival profiles in MM, indicating its clinical value for MM management.

## 1 INTRODUCTION

Multiple myeloma (MM) is a systemic malignant disease of the blood characterized by the production of nonfunctional intact immunoglobulins or immunoglobulin chains as well as the uncontrolled proliferation of monoclonal plasma cells in the bone marrow.^1^ According to the global statistics report, MM is the third most commonly occurred hematological malignancy, accounting for approximately 1% of all cancer cases, and its incidence has undergone an increment in recent decades.^2^ The common treatment approaches for MM consist of chemotherapy, autologous stem cell transplants, targeted therapy, and immunotherapy; however, the treatment efficacy is limited and the drug resistance is increasing due to the various chromosomal abnormalities, contributing to poor treatment response and undesirable survival profiles in MM patients.^3^, ^4^, ^5^, ^6^ Therefore, discovering novel prognostic biomarker is a necessity for MM management, which can help to estimate treatment response and predict prognosis in MM patients.

Long non‐coding RNA (lncRNA) is one type of RNA longer than 200 nucleotides in length with limited protein‐coding ability, and accumulating evidence has been reported that lncRNA is implicated in the tumorigenesis of various cancers.^7^ Among the discovered carcinogenic lncRNAs, lncRNA nuclear paraspeckle assembly transcript 1 (lncRNA NEAT1) is upregulated and serve as an oncogene during the onset and progression of various hematopoietic malignancies, such as myeloid leukemia and lymphoblastic leukemia.^8^, ^9^, ^10^, ^11^ As for in MM, mechanically, one cellular experiment demonstrates that lncRNA NEAT1 knockdown inhibits cell proliferation, but promotes cell‐cycle arrest and apoptosis via regulating PI3K/AKT pathway.^12^ Another one indicates that lncRNA NEAT1 promotes M2 macrophage polarization, hence accelerating MM progression.^13^ In addition, based on miRanda database analysis and previous studies, one of lncRNA NEAT1 target microRNAs is microRNA‐125a (miRNA‐125a), and miRNA‐125a is shown to suppress MM progression.^14^, ^15^, ^16^ According to these evidences and the result of our preliminary study with small sample size, which indicated that lncRNA NEAT1 was upregulated in MM patients compared with healthy controls, the hypothesis was raised that lncRNA NEAT1 was involved in MM development and prognosis via interaction with miR‐125a; however, the related research is limited. Therefore, we performed the present study to explore the association of lncRNA NEAT1 with MM risk and further investigate its correlation with clinical features, treatment response, survival profiles, and its interaction with miR‐125a in MM patients.

## 2 METHODS

### 2.1 Participants

From January 2016 to June 2019, 114 de novo MM patients treated in Cancer Hospital of the University of Chinese Academy of Sciences were consecutively enrolled in this prospective study. The inclusion criteria were as follows: (a) newly diagnosed as MM according to the “International Myeloma Working Group updated criteria for the diagnosis of multiple myeloma”^17^; (b) identified as primary symptomatic MM (primary MM patients with clonal bone marrow plasma cells ≥ 10% or biopsy‐proven bony or extramedullary plasmacytoma with the presence of hyper‐calcemia, renal failure, anemia, and bone lesion)^1^; (c) aged 18‐80 years; (d) not complicated with other malignancies; and (e) able to be followed up regularly. The exclusion criteria were as follows: (a) secondary (patients diagnosed as other malignancies before) or mixed (patients diagnosed as both MM and other malignancies) MM; (b) history of radiation and chemotherapy; (c) history of other hematopoietic diseases, lymphoid tissue diseases, or solid tumors; and (d) pregnant or lactating women. In addition, 30 healthy bone marrow donors who were admitted to our hospital and donated their bone marrow were recruited as controls in this study during the same period. This study was approved by the Institutional Review Board of Cancer Hospital of the University of Chinese Academy of Sciences. All patients and healthy donors provided the written informed consents before recruitment.

### 2.2 Clinical data collection

Patients’ age, gender, immunoglobulin subtype, bone lesion status, renal impairment status, Durie‐Salmon stage, International Staging System (ISS) stage, biochemical indexes, and cytogenetics status were recorded post‐diagnostic examinations. The Durie‐Salmon stage was assessed according to the criteria of Durie‐Salmon stage system for MM.^18^ The ISS stage was evaluated referring to the criteria of ISS for MM.^19^

### 2.3 Sample collection and determination

Before therapy, bone marrow samples of patients were collected; also, the bone marrow samples were collected from healthy donors after recruitment. For the isolation of plasma cells, all bone marrow samples were treated by density‐gradient centrifugation and purified by CD138‐coated magnetic beads (Miltenyi Biotec). Subsequently, reverse transcription quantitative polymerase chain reaction (RT‐qPCR) was performed to detect the relative expressions of lncRNA NEAT1 and miR‐125a in the plasma cells. Total RNA was extracted from plasma cells using TRIzol™ Reagent (Thermo Fisher Scientific) and then reversely transcribed using PrimeScript™ RT reagent Kit (Perfect Real Time) (Takara). Following that, qPCR was performed using SYBR^®^ Premix DimerEraser™ (Takara) to quantify lncRNA NEAT1 and miR‐125a expressions. In addition, the expressions of lncRNA NEAT1 and miR‐125a were calculated using 2^−ΔΔ^ *^C^* ^t^ method with GAPDH and U6 as internal references, respectively. Primers were listed in the Table S1.

### 2.4 Response and survival evaluation

Patients’ therapy in this study was not intervened, which was decided by attending physician based on patients’ clinical conditions in accordance with clinical practice guidelines.^20^ The patients’ response to the induction therapy was evaluated in line with the criteria recommended by NCCN clinical practice guidelines in Oncology: Multiple Myeloma (2015.V4), which included complete response (CR), very good partial response (VGPR), and partial response (PR). Overall response rate (ORR) was defined as CR + VGPR + PR. Patients were followed up by telephone or clinical visit until 2019/6/30, during which, patients’ survival status was documented for the assessment of progression‐free survival (PFS) and overall survival (OS). The PFS was defined as the duration from initial treatment to disease progression or death, and the OS was defined as the duration from initial treatment to death. The patients not known whether the disease had progressed or whether they had died at the last follow‐up date were censored on the date of last visit or the date last known to be alive.

### 2.5 Statistical analysis

SPSS 22.0 (IBM) and GraphPad Prism 6.01 (GraphPad Software) were used for statistical analyses and figures making. Data were described as mean and standard deviation (SD), median and interquartile range (IQR), or number (percentage). Comparison of continuous variables and ordered categorical variables between two groups was determined by Wilcoxon's rank sum test. Comparison of unordered categorical variables between two groups was determined by chi‐square test. The correlation analysis was determined by Spearman's rank correlation test. Receiver operating characteristic (ROC) curve and the area under curve (AUC) with 95% confidence interval (CI) were used to evaluate the value of variables in differentiating different subjects. PFS and OS were presented using Kaplan‐Meier curves, and the difference of PFS and OS between two groups was determined by the log‐rank test. *P* value < .05 was considered as statistically significant.

## 3 RESULTS

### 3.1 Clinical characteristics in MM patients

The mean age of MM patients was 54.7 ± 8.6 years, and there were 46 (40.4%) females and 68 (59.6%) males. As for immunoglobulin subtype, the number of MM patients with IgG, IgA, and other subtypes were 62 (54.4%), 27 (23.7%), and 25 (21.9%), respectively. There were 15 (13.2%) MM patients at Durie‐Salmon stage II and 99 (86.8%) MM patients at Durie‐Salmon stage III. Furthermore, the number of patients at ISS stages I, II, and III was 29 (25.4%), 27 (23.7%), and 58 (50.9%), respectively. More detailed information of clinical characteristics of MM patients was shown in Table 1.

**Table 1:** Clinical characteristics of MM patients

Items	MM patients (N = 114)
Age (years), mean ± SD	54.7 ± 8.6
Gender, No. (%)	
Female	46 (40.4)
Male	68 (59.6)
Immunoglobulin subtype, No. (%)	
IgG	62 (54.4)
IgA	27 (23.7)
Others	25 (21.9)
Bone lesion, No. (%)	
No	27 (23.7)
Yes	87 (76.3)
Renal impairment, No. (%)	
No	68 (59.6)
Yes	46 (40.4)
Durie‐Salmon stage, No. (%)	
II	15 (13.2)
III	99 (86.8)
ISS stage, No. (%)	
I	29 (25.4)
II	27 (23.7)
III	58 (50.9)
Biochemical indexes, median (IQR)	
Hb (g/L)	98.0 (82.0‐113.0)
Calcium (mg/dL)	9.7 (8.4‐11.2)
Scr (mg/dL)	1.8 (1.4‐2.2)
ALB (g/L)	34.0 (29.0‐38.0)
β2‐MG (mg/L)	5.6 (3.0‐10.0)
LDH (U/L)	210.8 (175.0‐249.5)
t(4; 14), No. (%)	
No	99 (86.8)
Yes	15 (13.2)
t(14; 16), No. (%)	
No	109 (95.6)
Yes	5 (4.4)
Del(17p), No. (%)	
No	100 (87.7)
Yes	14 (12.3)

Note

Continuous variables were expressed as mean ± SD or median (IQR). Categorical variables were expressed as count and percentage.

Abbreviations: ALB, albumin; Hb, hemoglobin; IgA, immunoglobulin A; IgG, immunoglobulin G; IQR: interquartile range; ISS, International Staging System; LDH, lactate dehydrogenase; MM, multiple myeloma; Scr, serum creatinine; SD, standard deviation; β2‐MG, beta‐2 microglobulin.

### 3.2 lncRNA NEAT1 expression between MM patients and health donors

**Figure 1:**
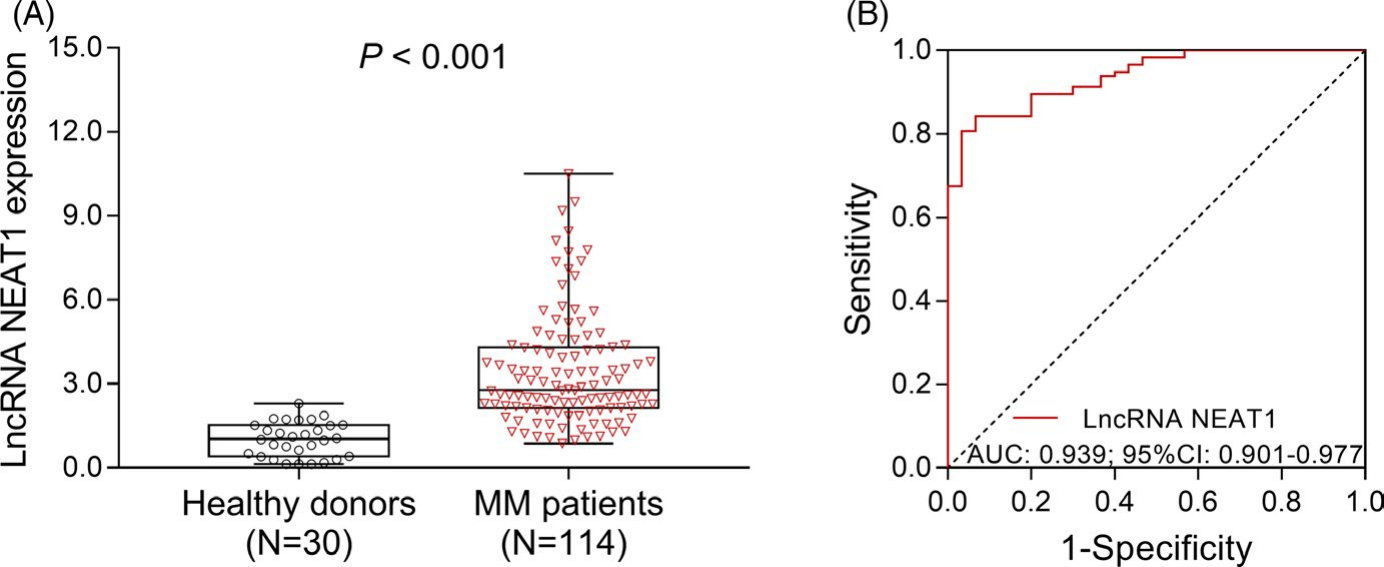
lncRNA NEAT1 in MM patients and healthy donors. Comparison of lncRNA NEAT1 between MM patients and healthy donors (A). The performance of lncRNA NEAT1 in distinguishing MM patients from healthy donors (B). MM, multiple myeloma; lncRNA NEAT1, long non‐coding RNA nuclear paraspeckle assembly transcript 1; AUC: area under curve; CI: confidence interval

lncRNA NEAT1 expression was increased in MM patients (2.787 [2.132‐4.293]) compared with healthy donors (1.029 [0.402‐1.524]) (*P* < .001) (Figure 1A). ROC analysis exhibited that lncRNA NEAT1 presented with excellent value in distinguishing MM patients from healthy donors (AUC: 0.939, 95% CI: 0.901‐0.977) (Figure 1B). These data suggested that high lncRNA NEAT1 expression was associated with increased MM risk.

### 3.3 Correlation of lncRNA NEAT1 with immunoglobulin subtype and stages in MM patients

**Figure 2:**
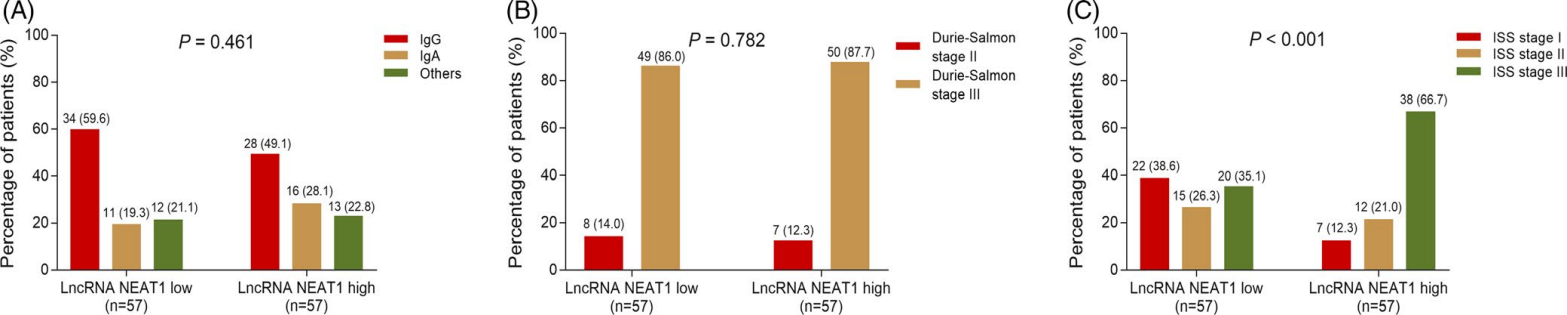
Comparison of immunoglobulin subtype and stages between high lncRNA NEAT1 MM patients and low lncRNA NEAT1 MM patients. Correlation of lncRNA NEAT1 with immunoglobulin subtype (A). Correlation of lncRNA NEAT1 with Durie‐Salmon stage (B). Correlation of lncRNA NEAT1 with ISS stage (C). MM, multiple myeloma; lncRNA NEAT1, long non‐coding RNA nuclear paraspeckle assembly transcript 1; ISS, International Staging System

According to the median of lncRNA NEAT1 expression in MM patients, all MM patients were divided into the low lncRNA NEAT1 patients (n = 57) and high lncRNA NEAT1 patients (n = 57). Among low lncRNA NEAT1 patients, there were 34 (59.6%) patients with lgG, 11 (19.3%) patients with lgA, and 12 (21.1%) patients with others; among high lncRNA NEAT1 patients, there were 28 (49.1%) patients with lgG, 16 (28.1%) patients with lgA, and 13 (22.8%) patients with others, and there was no difference of immunoglobulin subtype between low lncRNA NEAT1 patients and high lncRNA NEAT1 patients (*P* = .461) (Figure 2A). Furthermore, among low lncRNA NEAT1 patients, there were 8 (14.0%) patients at Durie‐Salmon stage II and 49 (86.0%) patients at Durie‐Salmon stage III; among high lncRNA NEAT1 patients, there were 7 (12.3%) patients at Durie‐Salmon stage II and 50 (87.7%) patients at Durie‐Salmon stage III, and there existed no difference of Durie‐Salmon stage between low lncRNA NEAT1 patients and high lncRNA NEAT1 patients (*P* = .782) (Figure 2B). In addition, among low lncRNA NEAT1 patients, the number of patients at ISS stages I, II, and III was 22 (38.6%), 15 (26.3%), and 20 (35.1%) respectively; among high lncRNA NEAT1 patients, the number of patients at ISS stages I, II, and III was 7 (12.3%), 12 (21.0%), and 38 (66.7%), respectively, and high lncRNA NEAT1 patients have increased ISS stage compared to low lncRNA NEAT1 patients (*P* < .001) (Figure 2C).

### 3.4 Correlation of lncRNA NEAT1 with core cytogenetics in MM patients

**Figure 3:**
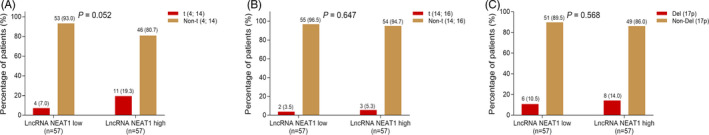
Comparison of cytogenetics between high lncRNA NEAT1 MM patients and low lncRNA NEAT1 MM patients. Correlation of lncRNA NEAT1 with t(4;14) occurrence (A). Correlation of lncRNA NEAT1 with t(14;16) occurrence (B). Correlation of lncRNA NEAT1 with Del(17p) occurrence (C). MM, multiple myeloma; lncRNA NEAT1, long non‐coding RNA nuclear paraspeckle assembly transcript 1

The number of patients with t(4;14) was 4 (7.0%) in low lncRNA NEAT1 patients and 11 (19.3%) in high lncRNA NEAT1 patients, and there was no difference of t(4;14) occurrence between low lncRNA NEAT1 patients and high lncRNA NEAT1 patients (*P* = .052) (Figure 3A). The number of patients with t(14; 16) was 2 (3.5%) in low lncRNA NEAT1 patients and 3 (5.3%) in high lncRNA NEAT1 patients, and there was no difference of t(14; 16) occurrence between high lncRNA NEAT1 patients and low lncRNA NEAT1 patients (*P* = .647), either (Figure 3B). Besides, the number of patients with Del (17p) was 6 (10.5%) in low lncRNA NEAT1 patients and 8 (14.0%) in high lncRNA NEAT1 patients, and there was no difference of Del (17p) occurrence between high lncRNA NEAT1 patients and low lncRNA NEAT1 patients (*P* = .568) (Figure 3C).

### 3.5 Correlation of lncRNA NEAT1 with biochemical indexes in MM patients

The level of hemoglobin (Hb), calcium, serum creatinine (Scr), albumin (ALB), beta‐2 microglobulin (β2‐MG), and lactate dehydrogenase (LDH) was 97.0 (75.5‐114.0) g/L, 9.7 (8.3‐11.7) mg/dL, 1.8 (1.4‐2.3) mg/dL, 35.0 (31.0‐39.0) g/L, 4.1 (2.5‐6.7) mg/L, and 192.1 (169.9‐235.8) U/L in low lncRNA NEAT1 patients, and 100.0 (84.0‐113.0) g/L, 9.7 (8.5‐11.7) mg/dL, 2.0 (1.5‐2.7) mg/dL, 33.0 (28.0‐37.0) g/L, 7.2 (4.1‐11.8) mg/L, and 221.4 (192.1‐292.8) U/L in high lncRNA NEAT1 patients, respectively (Table 2). lncRNA NEAT1 was positively associated with β2‐MG (*P* = .002) and LDH (*P* = .002); however, there was no correlation of lncRNA NEAT1 with Hb (*P* = .431), calcium (*P* = .807), Scr (*P* = .212), or ALB (*P* = .078) in MM patients (Table 2).

**Table 2:** Comparison of biochemical indexes between high lncRNA NEAT1 patients and low lncRNA NEAT1 patients

Biochemical indexes	lncRNA NEAT1		*P* value
	Low expression patients (n = 57)	High expression patients (n = 57)	
Hb (g/L), median (IQR)	97.0 (75.5‐114.0)	100.0 (84.0‐113.0)	.431
Calcium (mg/dL), median (IQR)	9.7 (8.3‐11.7)	9.7 (8.5‐11.7)	.807
Scr (mg/dL), median (IQR)	1.8 (1.4‐2.3)	2.0 (1.5‐2.7)	.212
ALB (g/L), median (IQR)	35.0 (31.0‐39.0)	33.0 (28.0‐37.0)	.078
β2‐MG (mg/L), median (IQR)	4.1 (2.5‐6.7)	7.2 (4.1‐11.8)	.002
LDH (U/L), median (IQR)	192.1 (169.9‐235.8)	221.4 (192.1‐292.8)	.002

Note

Comparison was determined by Wilcoxon's rank sum test.

Abbreviations: ALB, albumin; Hb, hemoglobin; IQR: interquartile range; LDH, lactate dehydrogenase; Scr, serum creatinine; β2‐MG, Beta‐2‐microglobulin.

### 3.6 Correlation of lncRNA NEAT1 with CR and ORR in MM patients

**Figure 4:**
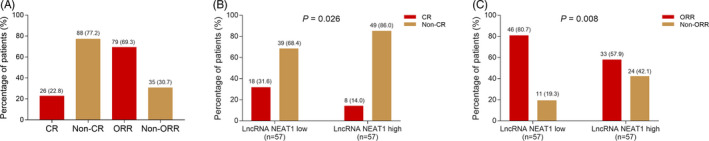
Comparison of CR/ORR between high lncRNA NEAT1 MM patients and low lncRNA NEAT1 MM patients. The percentage of CR, non‐CR, ORR, and non‐ORR patients (A). Correlation of lncRNA NEAT1 with CR (B). Correlation of lncRNA NEAT1 with ORR (C). MM, multiple myeloma; lncRNA NEAT1, long non‐coding RNA nuclear paraspeckle assembly transcript 1; CR, complete remission; ORR, overall remission rate

There were 26 (22.8%) CR patients, 88 (77.2%) non‐CR patients, 79 (69.3%) ORR patients, and 35 (30.7%) non‐ORR patients in MM patients (Figure 4A). The number of CR and non‐CR patients was 18 (31.6%) and 39 (68.4%) in low lncRNA NEAT1 patients, respectively, and was 8 (14.0%) and 49 (86.0%) in high lncRNA NEAT1 patients, respectively (Figure 4B). The number of ORR and non‐ORR patients was 46 (80.7%) and 11 (19.3%) in low lncRNA NEAT1 patients, respectively, and was 33 (57.9%) and 24 (42.1%) in high lncRNA NEAT1 patients, respectively (Figure 4C). Further analysis revealed that lncRNA NEAT1 was negatively associated with CR (*P* = .026) and ORR (*P* = .008) in MM patients (Figure 4B‐C).

### 3.7 Correlation of lncRNA NEAT1 with PFS and OS in MM patients

**Figure 5:**
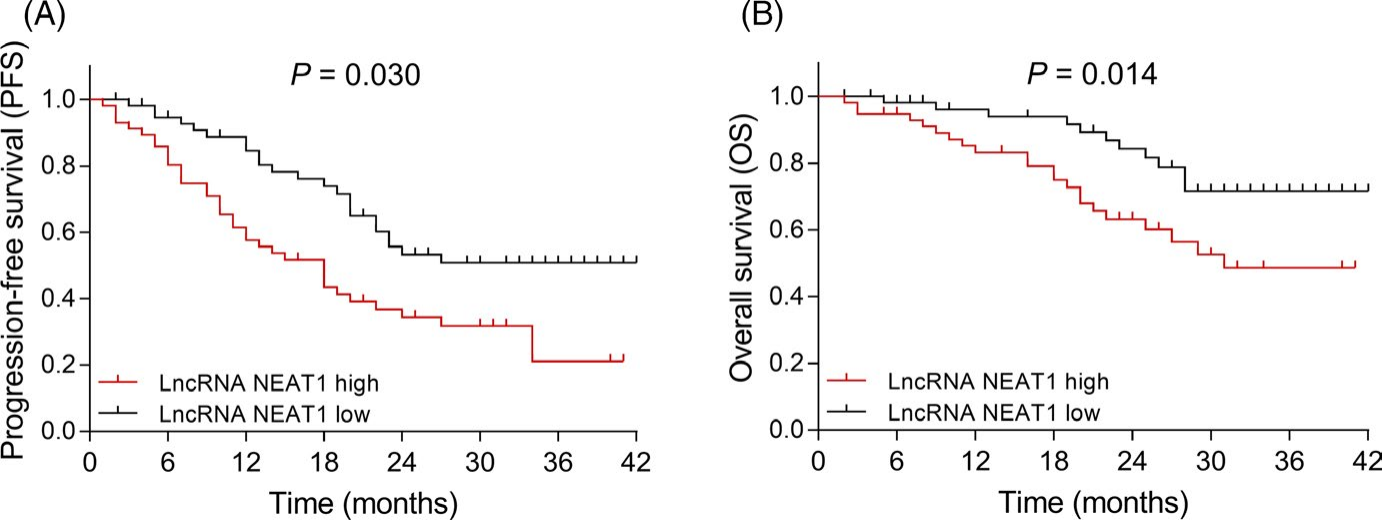
Comparison of PFS/OS between high lncRNA NEAT1 MM patients and low lncRNA NEAT1 MM patients. Comparison of PFS between high lncRNA NEAT1 MM patients and low lncRNA NEAT1 MM patients (A). Comparison of OS between high lncRNA NEAT1 MM patients and low lncRNA NEAT1 MM patients (B). MM, multiple myeloma; lncRNA NEAT1, long non‐coding RNA nuclear paraspeckle assembly transcript 1; PFS, progression‐free survival; OS, overall survival

PFS was decreased in high lncRNA NEAT1 patients compared with low lncRNA NEAT1 patients (*P* = .030) (Figure 5A). Similarly, OS was also reduced in high lncRNA NEAT1 patients compared with low lncRNA NEAT1 patients (*P* = .014) (Figure 5B).

### 3.8 Correlation of miR‐125a with lncRNA NEAT1, clinical features and prognosis in MM patients

**Figure 6:**
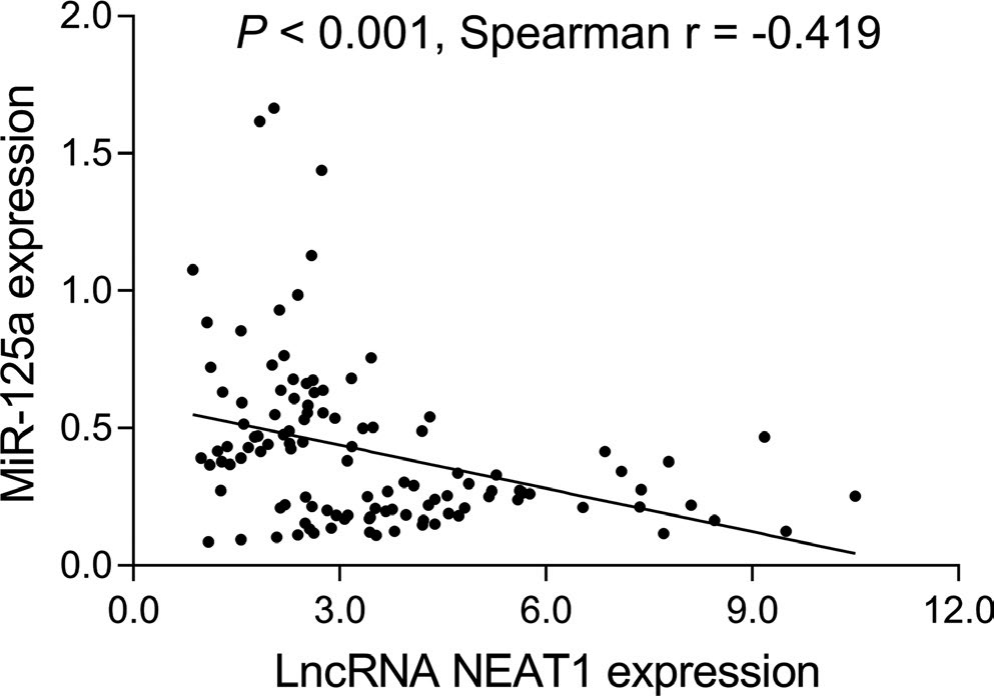
lncRNA NEAT1 correlated with miR‐125a in MM patients. MM, multiple myeloma; lncRNA NEAT1, long non‐coding RNA nuclear paraspeckle assembly transcript 1; miR‐125a, microRNA‐125a

**Figure 7:**
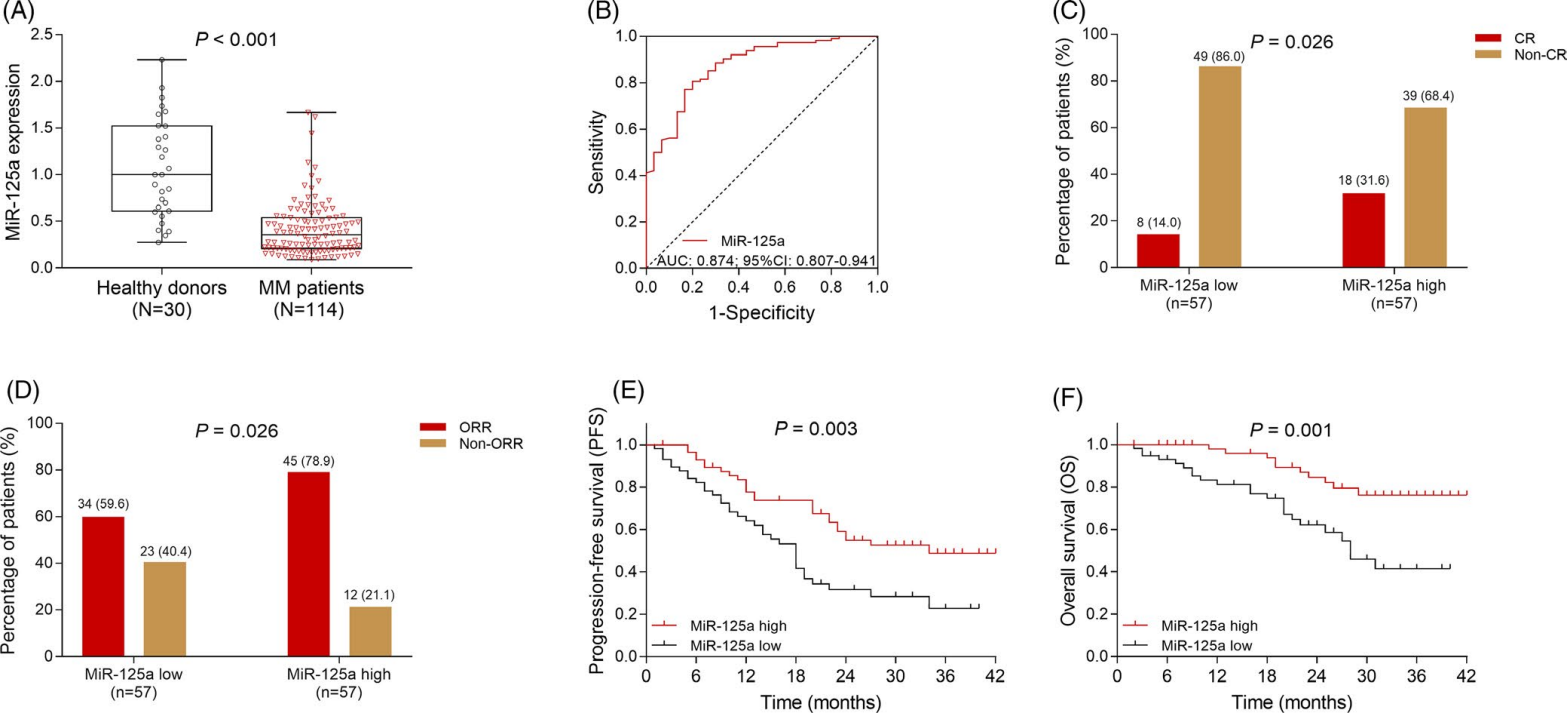
The value of MiR‐125a in predicting MM risk and its association with clinical features and prognosis in MM patients. Comparison of miR‐125a between MM patients and healthy donors (A). The performance of miR‐125a in distinguishing MM patients from healthy donors (B). Correlation of miR‐125a with CR (C), ORR (D) in MM patients. Correlation of miR‐125a with PFS (E) and OS (F) in MM patients. MM, multiple myeloma; miR‐125a, microRNA‐125a; CR, complete remission; ORR, overall remission rate; PFS, progression‐free survival; OS, overall survival; AUC: area under curve; CI: confidence interval

lncRNA NEAT1 was negatively associated with miR‐125a in MM patients (*r* = −.419, *P* < .001) (Figure 6). Furthermore, miR‐125a expression was decreased in MM patients compared with healthy donors (*P* < .001) (Figure 7A). ROC analysis revealed that miR‐125a presented with good value in distinguishing MM patients from healthy donors (AUC: 0.874, 95% CI: 0.807‐0.941) (Figure 7B). Further analysis detecting the association of miR‐125a with clinical features in MM patients indicated that miR‐125a was negatively associated with ISS stage (*P* = .001), β2‐MG (*P* = .002), and LDH (*P* = .002), while there was no association of miR‐125a with immunoglobulin subtype (*P* = .445), Durie‐Salmon stage (*P* = .406), t(4;14) (*P* = .406) t(14;16) (*P* = .170), Del (17p) (*P* = .087), Hb (*P* = .363), calcium (*P* = .080), Scr (*P* = .270), or ALB (*P* = .197) (Table 3). In addition, the correlation of miR‐125a with prognosis in MM patients was determined, and we found that miR‐125a was positively associated with CR (*P* = .026) (Figure 7C) and ORR (*P* = .026) (Figure 7D) in MM patients. Besides, PFS (*P* = .003) (Figure 7E) and OS (*P* = .001) (Figure 7F) were increased in miR‐125a high patients compared to miR‐125a low patients.

**Table 3:** Comparison of clinical characteristics between high miR‐125a patients and low miR‐125a patients

Items	MiR‐125a		*P* value
	Low expression patients (n = 57)	High expression patients (n = 57)	
Immunoglobulin subtype, No. (%)			
IgG	28 (49.1)	34 (59.6)	.445
IgA	14 (24.6)	13 (22.9)	
Others	15 (26.3)	10 (17.5)	
Durie‐Salmon stage, No. (%)			
II	6 (10.5)	9 (15.8)	.406
III	51 (89.5)	48 (84.2)	
ISS stage, No. (%)			
I	9 (15.8)	20 (35.1)	.001
II	10 (17.5)	17 (29.8)	
III	38 (66.7)	20 (35.1)	
t(4; 14), No. (%)			
No	48 (84.2)	51 (89.5)	.406
Yes	9 (15.8)	6 (10.5)	
t(14; 16), No. (%)			
No	53 (93.0)	56 (98.2)	.170
Yes	4 (7.0)	1 (1.8)	
Del(17p), No. (%)			
No	47 (82.5)	53 (93.0)	.087
Yes	10 (17.5)	4 (7.0)	
Biochemical indexes, median (IQR)			
Hb (g/L)	100.0 (82.0‐116.5)	97.0 (82.0‐11.0)	.363
Calcium (mg/dL)	9.5 (8.0‐10.6)	10.0 (8.8‐11.7)	.080
Scr (mg/dL)	1.9 (1.6‐2.7)	1.7 (1.3‐2.3)	.270
ALB (g/L)	34.0 (27.5‐37.5)	34.0 (29.5‐38.5)	.197
β2‐MG (mg/L)	6.6 (4.4‐11.7)	4.0 (2.3‐7.2)	.002
LDH (U/L)	221.4 (192.5‐281.6)	192.1 (167.6‐239.3)	.002

Note

Comparison was determined by chi‐square test or Wilcoxon's rank sum test.

Abbreviations: ALB, albumin; Hb, hemoglobin; IgA, immunoglobulin A; IgG, immunoglobulin G; IQR: interquartile range; ISS, International Staging System; LDH, lactate dehydrogenase; Scr, serum creatinine; β2‐MG, beta‐2 microglobulin.

## 4 DISCUSSION

In the present study, we found that (a) lncRNA NEAT1 was upregulated in MM patients compared with healthy donors, and presented excellent value in predicting MM risk. (b) lncRNA NEAT1 was positively correlated with ISS stage, β2‐MG, and LDH in MM patients. (c) lncRNA NEAT1 was negatively associated with prognosis in MM patients. (d) In MM patients, lncRNA NEAT1 was negatively associated with lncRNA NEAT1, and further analysis indicated that miR‐125a was associated with decreased MM risk, lower ISS stage, β2‐MG, LDH, and better prognosis.

lncRNA NEAT1 is an indispensable structural component of paraspeckles, which participates in the stress response, and has regulatory effect to control the progression of transcription, pre‐mRNA splicing, and nuclear mRNA editing via mediating exposure to stress events.^21^ In last decades, accumulating researches have disclosed the sectional pathophysiological relevance of lncRNA NEAT1 and reveal that lncRNA NEAT1 might function as an oncogenic gene in the pathology of various hematopoietic malignancies.^8^, ^9^, ^10^, ^11^ Regarding the involvement of lncRNA NEAT1 in MM, it is reported to be upregulated in BM samples of MM patients compared to healthy donors, and mechanically, its knockdown inhibits M2 macrophage polarization via dysregulating JAK2/STAT3 signaling, further accelerating MM development and progression.^13^ According to aforementioned evidence, lncRNA NEAT1 might present with clinical significance in MM management. We enrolled MM patients and healthy donors, whose BM samples were collected for detecting lncRNA NEAT1 expression, and found that lncRNA NEAT1 was upregulated in MM patients compared with healthy donors and present excellent value in distinguishing MM patients from healthy donors. This was consistent with the previous studies, suggesting that lncRNA NEAT1 was an oncogenic gene in MM pathology and has potential to be a biomarker for predicting MM risk.^12^, ^22^, ^23^

Subsequently, we observed that lncRNA NEAT1 was positively associated with ISS stage, β2‐MG, and LDH in MM patients, suggesting the correlation of lncRNA NEAT1 with poor systematic disease condition. The possible reasons might include that (a) according to the previous study, lncRNA NEAT1 promotes MM cell proliferation, but decreases cell‐cycle arrest via regulating PI3K/AKT signaling pathway, suggesting the accelerating role of AKIP1 in MM progression. Therefore, patients with high lncRNA NEAT1 expression presented poor systematic disease condition.^12^ (b) Based on the previous studies, the downstream targeted genes of lncRNA NEAT1 is involved in the DNA repair machinery; therefore, lncRNA NEAT1 dysregulation is associated with massive DNA damage, further leading to diverse genetic abnormalities, which lead to the high risk of MM progression.^21^, ^23^ (c) According to the previous study, β2‐MG concentration was used to reflect the kidney function, and lncRNA NEAT1 aggravated lipopolysaccharide‐induced kidney injury via activating inflammation‐related pathway (such as NF‐κB pathway); therefore, lncRNA NEAT1 might be associated with level of β2‐MG via inducing renal function.^24^, ^25^ (d) Based on the previous evidence that elevated LDH is correlated with increased level of beta‐2 microglobulin, lncRNA NEAT1 might be correlated with LDH via affecting with β2‐MG.^26^

Regarding the predictive role of lncRNA NEAT1 in treatment response and survival profiles in MM, there is still no research reported yet. Therefore, we assessed the treatment response to the induction therapy and calculated the survival data in MM patients. Following that, the related analysis indicated that lncRNA NEAT1 was negatively associated with CR, ORR, PFS, and OS in MM patients, suggesting the potential of lncRNA NEAT1 as a prognostic biomarker in MM. The possible reason might be that according to the prior studies, lncRNA NEAT1 is involved in p53‐dependent DNA damage response network, which has connection with increased chemotherapy resistance in MM patients.^21^, ^27^ Furthermore, considering our data that patients with high lncRNA NEAT1 expression presented poor systematic disease condition, MM patients with high lncRNA NEAT1 had poor treatment response and unfavorable survival.

lncRNAs compete with other RNA molecules to bind specific miRNA, and miRNA‐medicated competing endogenous regulatory mechanisms are reported to play essential role in pathogenesis of malignancies.^28^, ^29^ There exists evidence that lncRNA NEAT1 facilitates disease progression via interaction with miR‐125a; moreover, miR‐125a serves as a potential malignancy suppressor and targets MM‐related oncogenic genes, further suppressing MM progression.^14^, ^15^, ^30^ Therefore, we speculated that lncRNA NEAT1 might be implicated in the MM pathology via suppressing miR‐125a. In order to further investigate the regulatory role of lncRNA NEAT1 in MM, we explored the association of lncRNA NEAT1 with miR‐125a in MM and observed that there existed negative correlation between lncRNA NEAT1 and miR‐125a. In addition, miR‐125a was associated with decreased MM risk, and presented negative correlation with ISS stage, β2‐MG, LDH, unfavorable treatment response, and survival profiles. The possible reason might include that miR‐125a was a negative regulator of PI3K/Akt/mTOR and NF‐kB signaling pathways, which lncRNA NEAT1 had stimulating effect on; therefore, miR‐125a was negatively associated with MM risk, ISS stage, biochemical indexes (β2‐MG, LDH), and prognosis in MM patients.^31^, ^32^ The previous data implied the clinical significance of lncRNA NEAT1/miR‐125a complex in MM management.

However, there still exist some limitations in our present study. (a) Considering that this was a study with a small sample population, further studies with a larger sample size from multiple regions were needed for validation. (b) Our study did not explore the underlying mechanism of interaction between lncRNA NEAT1 and miR‐125a in MM, which needed to be investigated via cellular experiments. (c) As we excluded secondary or mixed MM, further studies were needed to investigate the association of lncRNA NEAT1 and miR‐125a with clinical indexes and prognosis in these patients.

In conclusion, lncRNA NEAT1 might interact with miR‐125a and serves as a novel biomarker for treatment response and survival profiles in MM, indicating its clinical value for MM management.

## CONFLICT OF INTEREST

The author declared no potential conflicts of interest with respect to the research, authorship, and/or publication of this article.

## Supporting information

Table S1
