# Supplementary material for: Long non‐coding RNA NEAT1 serves as a novel biomarker for treatment response and survival profiles via microRNA‐125a in multiple myeloma
Source: J Clin Lab Anal. 2020 Jul 1;34(9):e23399. doi: 10.1002/jcla.23399 (PMC7521229; doi:10.1002/jcla.23399)
Supplement: Supplementary file 1 — Table S1 [file JCLA-34-e23399-s001.docx]

**Supplementary table 1.** Primers used in RT-qPCR

| Items | Forward primer | | Reverse primer |
| --- | --- | --- | --- |
| LncRNA NEAT1 | | TGTCCCTCGGCTATGTCAGA | GAGGGGACGTGTTTCCTGAG |
| MiR-125a | ACACTCCAGCTGGGTCCCTGAGACCCTTTAAC | | TGTCGTGGAGTCGGCAATTC |
| GAPDH | TGACCACAGTCCATGCCATCAC | | GCCTGCTTCACCACCTTCTTGA |
| U6 | CTCGCTTCGGCAGCACATATACTA | | ACGAATTTGCGTGTCATCCTTGC |
